# New Pharmacological Approaches for the Treatment of Neurotrophic Keratitis

**DOI:** 10.3389/fphar.2022.796854

**Published:** 2022-03-22

**Authors:** Su Yin Koay, Daniel F. P. Larkin

**Affiliations:** Cornea and External Diseases Service, Moorfields Eye Hospital, London, United Kingdom

**Keywords:** neurotrophic keratitis, persistent epithelial defect, nerve growth factor, corneal ulcer, neurotization

## Abstract

Neurotrophic keratitis (NK) is a rare degenerative condition that is caused by damage to the trigeminal nerve, with partial or complete loss of corneal sensory innervation. The loss of innervation leads to impaired healing of corneal epithelium, which subsequently results in punctate epithelial erosions, persistent epithelial defects, corneal ulcers and corneal perforation. Management of NK is often supportive and aims to promote epithelial healing and prevent progression of disease. Multiple novel pharmacological approaches have been proposed to address the underlying pathophysiology of NK, which are discussed in this paper.

## Introduction

Neurotrophic keratitis (NK) is caused by damage to the trigeminal nerve, with partial or complete loss of corneal innervation and sensation. This leads to impairment in the sensory and trophic function of the corneal nerves, as well as reduction of both protective reflexes and trophic neuromodulators, with consequent breakdown of the corneal epithelium (Dua et al., 2018; Versura et al., 2018). It is a rare degenerative condition with an estimated prevalence of 1.6–4.2 cases per 10,000 persons (Mastropasqua et al., 2017). NK can occur as a result of congenital abnormalities, ocular pathology (most commonly herpetic infections), neurological conditions or surgery, and systemic conditions such as diabetes mellitus (Bonini et al., 2003). It may also occur due to direct damage to corneal nerve endings following chronic usage of certain eye drops such as timolol. (Table 1).

**TABLE 1:** Classified causes of neurotrophic keratopathy.

**Inherited and Congenital**
Familial dysautonomia (Riley Day)
Familial corneal anesthesia
Hereditary and sensory autonomic neuropathy types IV and V
Mobius syndrome
Goldenhar syndrome
**Ocular**
Herpes simplex keratitis
Herpes zoster keratitis
Corneal surgery (keratoplasty, refractive surgery)
Contact lens wear
Chemical or physical burns
Chronic ocular surface inflammation
Corneal dystrophies (granular, lattice)
Drug toxicity (timolol, diclofenac)
Orbital neoplasia
**Neurological**
Traumatic/surgical resection of trigeminal nerve
Surgical ablation for trigeminal neuralgia
Compressive/infiltrative lesion, e.g. acoustic neuroma, aneurysm
Stroke
**Systemic**
Diabetes mellitus
Leprosy
Vitamin A deficiency

**FIGURE 1:**
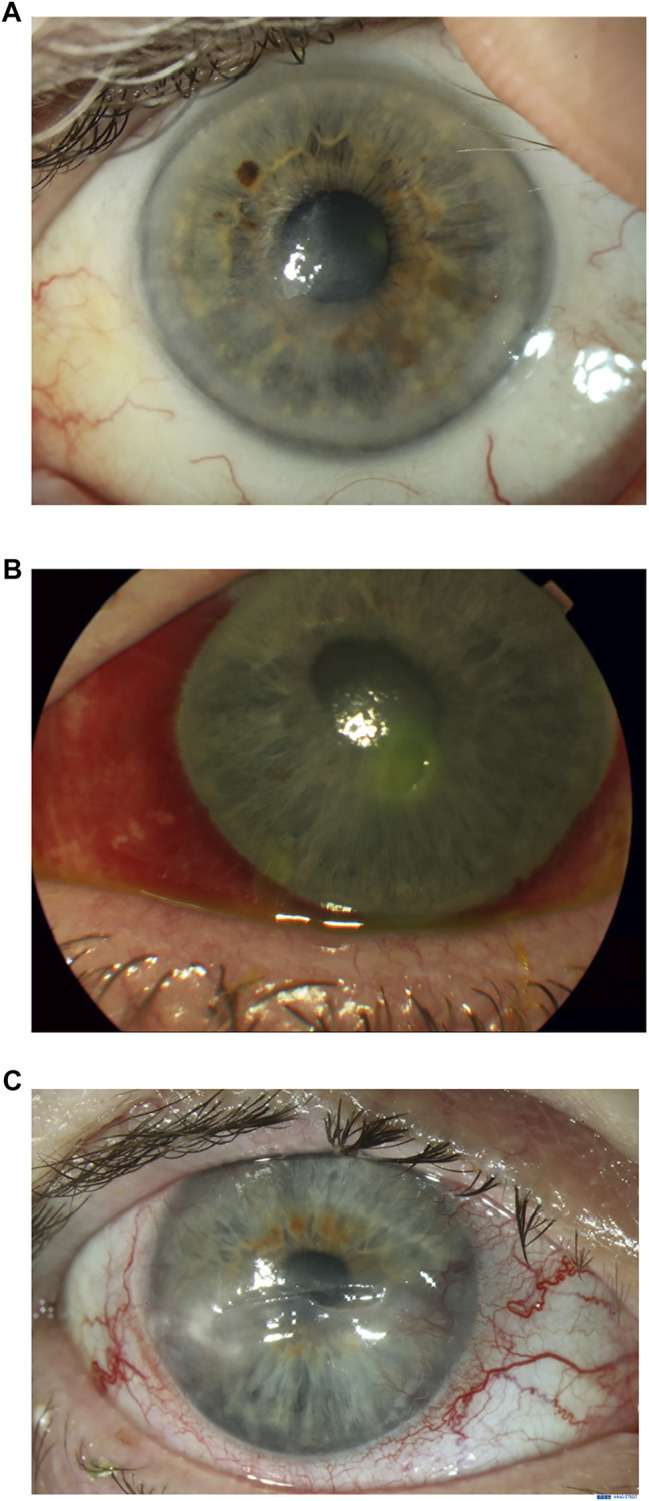
Clinical appearance of neurotrophic keratitis (NK) stages 1, 2 and 3. Figure 1A. Stage 1 NK: Superficial opacity and punctate corneal epitheliopathy. Figure 1B. Stage 2 NK: Persistent epithelial defect. Figure 1C. Stage 3 NK: Ulceration with thinning to mid-stroma.

NK is classified into three stages according to the Mackie classification (Bonini et al., 2003). In *Stage 1*, epithelial changes are seen. Clinical findings include epithelial hyperplasia and irregularity, punctate keratopathy, dellen, decreased tear break-up times, stromal scarring and superficial vascularization (Figure 1A). Persistent epithelial defects (PEDs) are the hallmark of *Stage 2*, these are usually oval in shape with a characteristic rolled and smooth edge (Figure 1B). Stromal edema, Descemet’s membrane folds or anterior chamber inflammatory cells may also be seen. In *Stage 3*, a corneal ulcer with stromal involvement is present (Figure 1C). This may progress to corneal melting and subsequent perforation.

The management of NK is challenging, and vision loss often occurs despite treatment to support epithelialization. Visual prognosis depends on the underlying etiology and the degree of corneal hypoanesthesia. This has led to the development of new medical treatments to treat this condition, which is the focus of this article. Surgical management is also briefly reviewed as this is often necessary when managing NK.

## Assessment of NK

**FIGURE 2:**
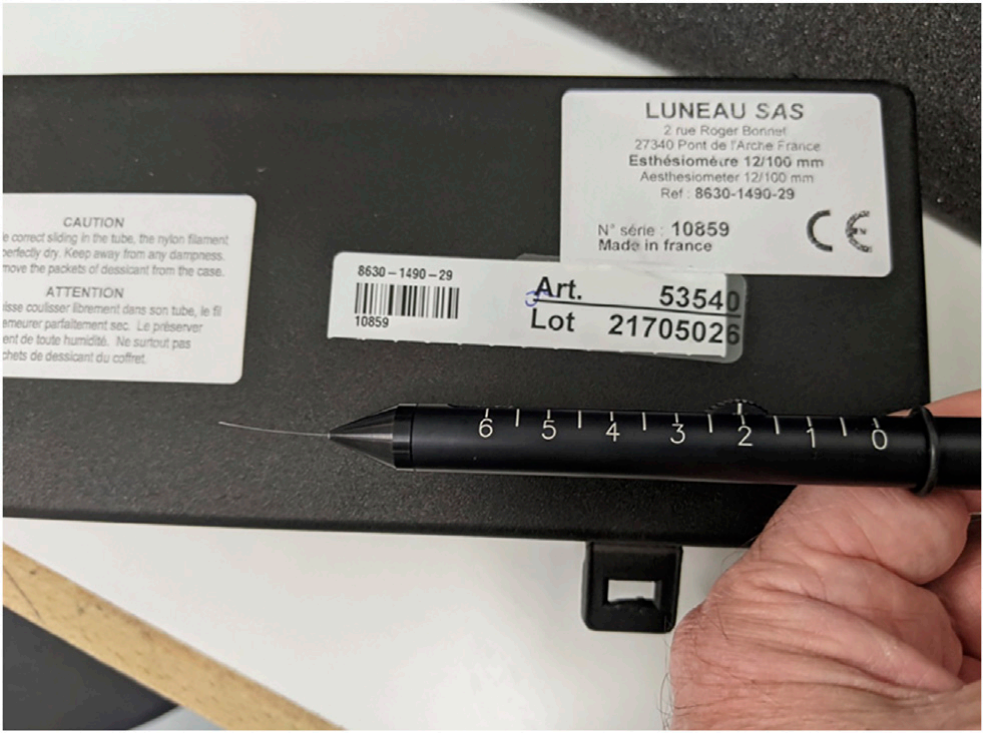
Cochet-Bonnet esthesiometer.

Corneal sensitivity can be measured qualitatively by touching all four quadrants of the cornea with a twisted cotton swab. Quantitative testing can be performed with the Cochet-Bonnet esthesiometer (Figure 2), which records the patient’s response or blink to a length of a protruding nylon filament (between 0 and 60 mm). The filament is used to touch the cornea, following which pressure is applied to induce a bend in the filament. The filament length is gradually reduced until the patient is able to report the filament touching the cornea. A normally innervated cornea usually detects touch at a filament length of 40 mm. A shorter filament length corresponds with poorer corneal sensation.

**FIGURE 3:**
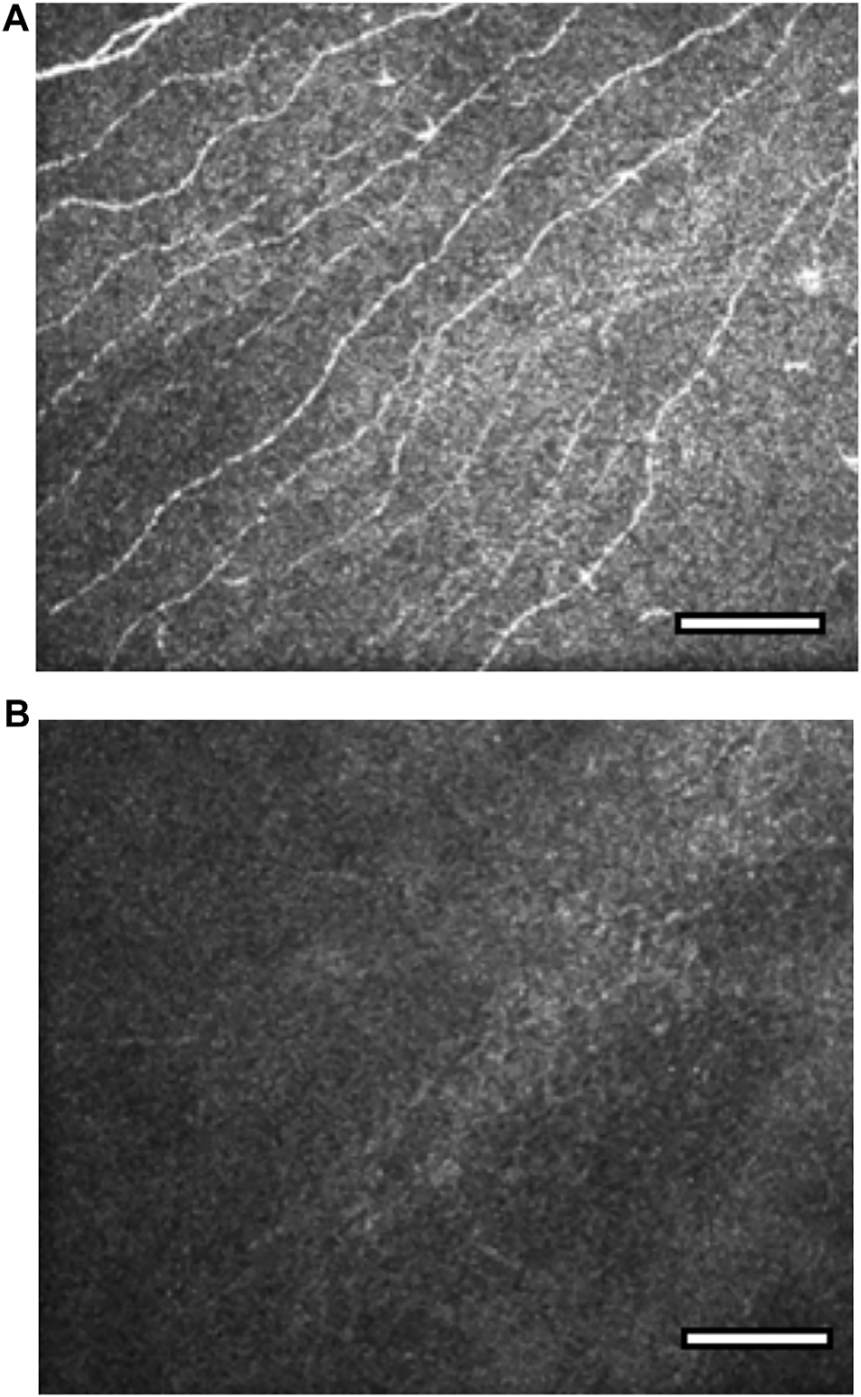
*In vivo* confocal microscopy images of the sub-basal nerve plexus in congenital corneal anaesthesia (CCA). Reproduced with permission from the British Journal of Ophthalmology and Guillon-Rolf et al. (Guillon-Rolf et al., 2020). Figure 3A. Sub-basal plexus in a normal cornea at depth 50 μm. Figure 3B. Absence of sub-basal nerve plexus nerves in a patient with congenital corneal anaesthesia at depth 50 μm.

Slit lamp examination with dilated fundoscopy may give clues to the etiology of the NK. In addition, it is also important to perform a full cranial nerve examination in all patients as the involvement of the other cranial nerves may allow localization of a neurological lesion. For example, involvement of the facial (VII) or vestibulocochlear (VIII) nerve may indicate a potential acoustic neuroma. *In vivo* confocal microscopy (IVCM) is not particularly helpful for diagnosis, but may have a role in longitudinal evaluation of patients in clinical trials of new treatments or neurotization surgery. Guillon-Rolf et al. demonstrated a statistically significant reduction in sub-basal nerve plexus nerve fiber density, fiber length and branch density with IVCM in patients with congenital corneal anesthesia (Figure 3). (Guillon-Rolf et al., 2020)

## Management of NK

The aim of management in NK is to prevent progression of disease (Lambiase et al., 1999). In Stage 1, management focuses on preventing breakdown of corneal epithelium. In addition, any eyelid position abnormalities (e.g., lagophthalmos) and potentially toxic topical medications should be identified and addressed.

The aims in stages 2 and 3 are to promote epithelial healing, and to prevent the development of infective keratitis or corneal melt (Semeraro et al., 2014a). Regular monitoring is essential as patients are often unaware of any worsening of symptoms.

### Conventional Medical Treatment

Ocular lubricants are used in all stages of NK, and promote epithelialization by reducing biomechanical shear forces and diluting pro-inflammatory mediators in the tear film (Dua et al., 2018). Frequent use of non-preserved ocular lubricants is encouraged in all stages of NK and include hypromellose, carbomer gels, hydroxypropylguar, hyaluronate and hyaluronate combinations (carboxymethylcellulose, polysaccharide, soybean with phospholipids, xanthan gum, trehalose).

When an epithelial defect is present, prophylactic topical antibiotics help to reduce the risk of infective keratitis.

Matrix metalloproteinases inhibitors such as oral tetracyclines, oral erythromycin and topical azithromycin restrict neutrophil collagenase and epithelial gelatinase gene expression, suppress alpha-1 antitrypsin degradation and scavenge reactive oxygen species (Dua et al., 2018). This can reduce the risk of corneal melt in patients with stage 3 NK (Trinh et al., 2021).

### Blood Product Derivatives

Autologous serum eye drops were first reported to be beneficial for Sjögren’s syndrome-related dry eye disease in 1984 (Fox et al., 1984). Serum eye drops contain growth factors (epidermal growth factor (EGF) and transforming growth factor (TGF)), vitamins (A, C), glucose, natural antimicrobials (surface IgA, defensins, lysozyme), and proteins involved in wound healing (fibronectin) (Rauz and Saw, 2010). These biological properties help to facilitate epithelialization by inducing cellular migration and adhesion (Matsumoto et al., 2004). Whilst there is a lack of randomized controlled trial data, serum eye drops have been reported to promote substantial improvement of PEDs unresponsive to conventional treatment within 7–28 days. (Tsubota et al., 1999; Lekhanont et al., 2013; Semeraro et al., 2014b; Azari and Rapuano, 2015; Shtein et al., 2020).

Matsumoto et al.(Matsumoto et al., 2004) used 20% autologous serum in 14 eyes with NK and achieved complete epithelial healing in 17.1 ± 8.0 days. Mean pretreatment corneal sensitivity was 11.8 ± 11.6 mm, which increased to 30.0 ± 22.9 mm after treatment with autologous serum. In addition, autologous serum eye drops have been shown to improve corneal nerve morphology with increased number, length, width, and density of subepithelial corneal nerves detected with IVCM post treatment (Rao et al., 2010; Aggarwal et al., 2015; Abedi and Hamrah, 2018).

Human umbilical cord serum has been shown to contain higher concentrations of EGF, NGF and TGF-β compared to peripheral blood serum (Yoon et al., 2006; Yoon et al., 2007). Vajpayee et al. (Vajpayee et al., 2003) reported that umbilical cord serum has been reported to lead to a faster median percentage decrease in the size of PEDs at 7, 14 and 21 days (*p* < 0.05) compared to peripheral blood serum. Yoon et al. (Yoon et al., 2007) treated 28 NK eyes with 20% umbilical serum reported complete epithelial defect healing in all eyes, with a mean healing time of 4.4 ± 4.0 weeks. Umbilical serum has also been shown to improve corneal nerve morphology (assessed with IVCM), with an increase of total nerve number and a decrease of nerve tortuosity (Giannaccare et al., 2017). Nevertheless, the small volumes obtained and the cost of preparation limits its widespread use (Dua et al., 2018).

Platelet rich plasma (PRP) and plasma rich in growth factors (PRGF) have been reported to be effective for promoting resolution of persistent epithelial defects which are unresponsive to conventional treatments (You et al., 2020). It contains a number of growth factors such as platelet-derived growth factor (PDGF), EGF, fibroblast growth factor (FGF), TGF, nerve growth factor (NGF), and insulin-like growth factor (IGF). (Mussano et al., 2016). In a cell culture inflammatory model where ocular surface fibroblasts treated with pro-inflammatory interleukin 1-beta (IL-1β) and tumor necrosis factor alpha (TNFα), PRGF appeared to exert more potent regenerative and anti-inflammatory effects compared to autologous serum (Anitua et al., 2016).

Wróbel-Dudzińska et al. treated 25 eyes with stages 2/3 NK with PRP, and reported complete healing of ulceration in 80% (*n* = 20), and considerable improvement in ulcer size in 16% (*n* = 4) (Wróbel-Dudzińska et al., 2018). Kim et al. reported that mean epithelial healing time of PEDs after infective keratitis was 10.09 ± 2.49 days (range 2–30 days) with PRP, compared to 17.83 ± 3.07 days (range 5–38 days) with autologous serum (Kim et al., 2012). An ongoing randomized non-masked trial (NCT03653650) aims to compare PRP + bandage contact lens; lubricants + bandage contact lens and lubricant ointment + eye patching in PEDs.

### Matrix Regenerating Agents

ReGeneraTing Agents (RGTAs) are biodegradable glucose-based polymers which are chemically bioengineered to form analogs of the extracellular matrix component. RGTAs mimic the action of heparan sulfates bound to extracellular matrix proteins, allowing them to form a bioskeleton scaffold that induces cell adhesion whilst providing proteolytic protection for the components involved in tissue healing (e.g., collagen, fibronectin, elastin) (Arvola et al., 2016) Their large molecular structure makes it unlikely to penetrate through the cornea. RGTA is usually instilled every 2-3 days, more frequent instillation is not recommended as it may negatively impact the healing process (Barritault et al., 2017).

Cacicol (Laboratoires Théa, Clermont-Ferrand, France) belongs to the RGTA family and is the first ophthalmic matrix therapy product. Results appear to be promising for the treatment of corneal ulcers resistant to conventional treatments. Aifa et al. administered RGTA for severe corneal neurotrophic ulcers, and achieved complete healing in eight of 11 eyes (73%) after a mean of 8.7 weeks (range 1–22 weeks) (Aifa et al., 2012). Mean ulcer area decreased from 11.12 to 6.37% (*p* = 0.048) in the first week, and to 1.56% (*p* = 0.005) at 1 month (Aifa et al., 2012). Guerra et al. reported corneal healing (defined as a decrease of the corneal ulcer area) in all 25 eyes with NK within an average of 4.1 ± 2.3 weeks. (Guerra et al., 2017). Cochener et al. administered RGTA to 20 patients (20 eyes) with stage 2/3 NK unresponsive to conventional treatment in a prospective observational study (Cochener et al., 2019). Total corneal healing was observed in 13 eyes (65%) within 1–3 months, with relapses reported in four eyes (20%) several months after cessation of treatment. A randomized multicenter double masked study (NCT01242839) which reported to have completed in June 2014 compared the healing rate of chronic corneal ulcers with the use of calcicol versus placebo drops, but results have not been published. The manufacturer ceased the production of calcicol in December 2019 for commercial reasons.

### Recombinant Human Nerve Growth Factor (rhNGF)

NGF is a neurotrophin that stimulates corneal re-innervation and healing after injury, induces epithelial cell proliferation and differentiation, maintains corneal epithelial stem cells, and can promote tear production (Deeks and Lamb, 2020). Cenegermin is a rhNGF produced in *Escherichia coli* which was developed following encouraging results of murine NGF in the treatment of NK (Lambiase et al., 1998; Bonini et al., 2000).

The REPARO study (Bonini et al., 2018) was a phase II European multicenter, randomized, double-masked, vehicle-controlled trial that assessed the safety and efficacy of rhNGF in stage 2/3 NK. 156 patients were randomized 1:1:1 to rhNGF 10 μg/ml, 20 μg/ml, or vehicle; drops were administered 6 times a day for 8 weeks. At week 4, 19.6% of vehicle-treated patients achieved corneal healing versus 54.9% receiving rhNGF 10 μg/ml (+35.3%; *p* < 0.001) and 58.0% receiving rhNGF 20 μg/ml (+38.4%, *p* < 0.001). At week 8, 43.1% of vehicle-treated patients achieved less than 0.5 mm lesion staining versus 74.5% receiving rhNGF 10 μg/ml (+31.4%; *p* = 0.001) and 74.0% receiving rhNGF 20 μg/ml (+30.9%; *p* = 0.002).

Median time to corneal healing was 56, 29 and 28 days in the vehicle, rhNGF 10 μg/ml and rhNGF 20 μg/ml groups. More than 96% of patients who healed after rhNGF treatment did not get a recurrence of the PED or corneal ulcer during the follow up period of 48–56 weeks. Treatment was well tolerated, with mild ocular side effects (conjunctival hyperaemia, photophobia and ocular pain) that did not require discontinuation of treatment. The authors hypothesized that these side effects may represent improvement of corneal sensitivity due to the therapeutic actions of rhNGF.

NGF0214 (Pflugfelder et al., 2020) was a further randomized, double-masked, vehicle-controlled trial of rhNGF performed in the United States. 48 patients with stage 2/3 NK were randomized 1:1 to rhNGF (cenegermin) 20 μg/ml or vehicle eye drops. After 8 weeks, 16 of 23 rhNGF-treated patients (69.6%) achieved less than 0.5 mm of lesion staining compared to seven of 24 vehicle-treated patients (29.2%) (+40.4%, *p* = 0.006). There were no statistically significant improvements in corneal sensitivity, but reflex tearing (a possible indication of corneal sensitivity and nerve function) exhibited trends favoring rhNGF treatment.

Neither the REPARO or NGF0214 study showed statistically significant improvements in visual acuity measures. It is however important to remember that in NK, visual acuity does not necessarily correlate with NK severity or healing status.

Following these two studies, cenegermin 0.002% ophthalmic solution (Oxervate; Dompé Farmaceutici SpA, Milan, Italy) received approval from the European Commission (EC) and the United States Food and Drug Administration. At the time of writing, it is commercially available in Europe, the United States, Switzerland, Israel, Canada and Australia. Despite EC approval, cenergermin is not available in the United Kingdom, following a decision on grounds of cost-effectiveness by the UK’s National Institute for Health and Care Excellence (NICE NC for H and CE, 2018).

Mastropasqua et al. evaluated the structural changes that occur as a result of rhNGF treatment in 18 patients with NK with IVCM (Mastropasqua et al., 2020). There was a significant increase in the mean sub-basal nerve density, diameter and number of nerve branches at weeks four and eight in comparison to baseline, with a nerve regeneration rate of 1079.1 ± 835 μm/mm^2^ at 4 weeks and 661.9 ± 835 μm/mm^2^ at 8 weeks. Despite documented regeneration of sub-basal nerve plexus following 8 weeks of treatment, the nerve fiber density, number of nerve branches and corneal nerve diameter were still statistically significantly less in the NK group compared to healthy controls. NCT04627571 (ongoing) is investigating the structural effects of rhNGF on the sub-basal corneal nerve density at 1 year in stage 2/3 NK.

Udonitrectag (also known as MT8 or REC 0559) is a low molecular weight, synthetic peptido-mimetic of NGF. MT8 binds the tropomyosin kinase A (TrkA) receptor, mimicking the anti-apoptotic and corneal trophic activity of NGF. It has been shown to improve healing of corneal epithelium and stroma in rabbit wound healing models. Its use in stage 2/3 NK is being investigated in a phase II multicenter, double masked, randomized trial (NCT04276558).

### Insulin

Insulin is a peptide that is closely related to insulin-like growth factor (IGF) and has been implicated in wound repair. Shanley et al. (Shanley et al., 2004) showed that exposure of corneal epithelium to insulin facilitated closure of *in vitro* small wounds through enhanced cell migration. The effect of topical insulin on corneal wound healing has been well reported in rodent models, and improves corneal re-epithelialization in diabetic rats (Zagon et al., 2007). The mechanisms are not clearly understood, but is thought to be due to restoration of corneal nerves and/or improved epithelial cell migration. (Wang et al., 2017). It is notable that insulin has been demonstrated to have a biological effect on corneal epithelialization, and diabetes mellitus is a recognized cause of neurotrophic keratitis.

Wang et al. (Wang et al., 2017) used topical insulin in six eyes with non-healing neurotrophic ulcers, and achieved complete corneal re-epithelialization in 7–25 days following initiation of treatment. Topical insulin was prepared by mixing regular insulin in artificial tears with a polyethylene glycol and propylene glycol base at a concentration of 1 unit per mL, and was prescribed 2–3 times daily.

In a prospective non-randomized study (Diaz-Valle et al., 2020), 21 eyes with PEDs were treated with topical insulin 1 unit per ml. 17 eyes (81%) had complete epithelial healing, mean time to reepithelization was 34.8 ± 29.9 days (median 23; range 7–114). Four eyes (19%) still had an epithelial defect but the mean PED size reduced by 91.5%.

A double masked, randomized trial (Fai et al., 2017) compared three concentrations of topical insulin (0.5 units, 1 unit and 2 units per drop) to placebo for the treatment of post-vitrectomy epithelial defects in 32 diabetic eyes. Topical insulin 0.5 units was superior to other concentrations and achieved a 100% healing rate within 72 h, compared with 62.5% for placebo, 75% for topical insulin 1 unit/drop, and 62.5% for insulin 2 units/drop. The authors postulate that the lack of benefit in using higher concentrations may be a result of greater toxicity and a decreased migration of epithelial cells during the healing process due to higher viscosity.

Topical insulin is well tolerated and does not cause ocular side effects when used in concentrations up to 100 units/ml (Bartlett et al., 1994). It is not absorbed systemically, and as such blood glucose levels and serum immunoreactive insulin levels are unchanged (Bartlett et al., 1994). Supported by evidence in the above reports, topical insulin is becoming more widely used in NK, and is manufactured locally rather than being produced commercially. An insulin concentration of 1 units/ml (Humulin S in Systane lubricant eye drops) has been used successfully in a number of patients in Moorfields Eye Hospital (S Ahmad, personal communication, 5 March 2022).

### Substance P and Insulin-Like Growth Factor 1 (IGF-1)

IGF-1 has been shown to be an important modulator of corneal wound healing, and acts synergistically with substance P to promote corneal epithelium wound healing (Nishida et al., 1996). The peptide SSSR, corresponding to a four-amino acid sequence in the C domain of IGF-1, is the minimal essential sequence for the synergistic stimulation with substance P of corneal epithelial migration (Yamada et al., 2006). The combination of the substance P-derived peptide (FGLM-amide) and SSSR likely targets corneal epithelial cells directly, bypassing nerve fibres and triggering epithelial migration through activation of intracellular signal transduction systems (Yamada et al., 2008).

Nakamura et al.(Yamada et al., 2008) treated 26 eyes with NK associated PEDs with a topical combination of substance P-derived peptide (FGLM-amide) and IGF-1-derived peptide (SSSR). Complete closure of epithelial defects was achieved in 19 of the 26 eyes (73%) within 4 weeks of treatment initiation. Of note, three of the seven non-responders had limbal stem cell deficiency, which may explain the lack of efficacy of the treatment given the role of limbal stem cells in epithelial healing.

Nishida et al.(Nishida et al., 2007) also used FGLM-amide and IGF-1 drops to treat nine eyes with PEDs from NK, and achieved complete epithelial healing in 89% at 28 days.

FGLM-amide and IGF-1 drops are not commercially available at present.

### Thymosin Beta 4 (Tβ4)

Tβ4 is a naturally‐occurring 43‐amino acid peptide that has been shown to promote corneal wound re-epithelialization, diminish inflammation, and inhibit apoptosis (Katzman and Jeng, 2014). Dunn et al. (Dunn et al., 2010) showed that Tβ4 drops promote healing in nine patients with chronic neurotrophic PEDs. Six patients with geographic defects showed rapid epithelial corneal healing, where four had complete healing and two had defects <0.1 mm. However the three patients with punctate epithelial defects did not show demonstrable changes in their clinical findings. Tβ4 eye drops were well tolerated by the patients, who all reported subjective improvement in ocular redness and foreign body sensation during the course of treatment (Dunn et al., 2010). Tβ4 drops are developed by RegeneRx Biopharmaceuticals, Inc. (Maryland, United States ) but are only in use for clinical trials at present (NCT03937882).

### Connexin43 (Cx43) Antisense Oligodeoxynucleotides (AsODN)

Transiently blocking the expression of the gap junction protein Cx43 using AsODNs or blocking hemichannels has been demonstrated to limit inflammation, edema and lesion spread and to provide improved healing in acute wound models (Grupcheva et al., 2012; Ormonde et al., 2012). Ormonde et al. (Ormonde et al., 2012) used Cx43-AsODN delivered in cold, thermoreversible Poloxamer407 gel (under an amniotic membrane graft or bandage contact lens) to treat PEDs from ocular burns. Ocular inflammation improved within 1-2 days, and all five eyes had complete and stable reepithelialization. To date there have been no studies reporting its use in NK, and Cx43 is not commercially available.

### Non-specific Ocular Surface Support

Therapeutic bandage contact lenses (BCLs) can be helpful in the treatment of NK, as they protect the advancing epithelial cells from being sloughed-off by the blinking eyelids, as well as by providing anesthetic relief (Katzman and Jeng, 2014). In a non-randomized comparative study, the healing time of NK related corneal ulcers was 10.80 ± 4.44 days with a soft silicone hydrogel BCL versus 46.70 ± 13.88 days in the control group (*p* < 0.05) (Sun et al., 2014). Scleral contact lenses allow a reservoir of fluid to be created between the contact lens and the ocular surface, which can also be helpful in NK (Grey et al., 2012). However there is a risk of infective keratitis with contact lens wear, even when prophylactic topical antibiotics are used (Saini et al., 2013; Zhu et al., 2019).

Punctal occlusion with silicone punctal plugs or surgical permanent occlusion is also often used to support the tear film in NK eyes (Sacchetti and Lambiase, 2014).

### Surgical Management

Tarsorrhaphy is a useful adjunct to promote healing of the ocular surface in stage 2/3 NK. This can either be temporary or permanent. Lateral tarsorrhaphies are usually permanent whilst central ones are typically temporary on account of the effect on vision in that eye. In some cases Botulinum toxin can be used instead of resorting to surgery, and has been shown to be effective for inducing temporary ptosis to protect the cornea (Kirkness et al., 1988). This has the advantage over central tarsorrhaphy of avoiding any surgical damage to the lid margin, instillation of eye drops is easy for patients and the ptosis reverses over 4–6 weeks.

Amniotic membrane (AM) transplantation is a form of ocular surface reconstruction which has been shown to be effective in the management of NK (Chen et al., 2000; Khokhar et al., 2005). AM contains growth factors such as NGF, keratinocyte growth factor, and hepatocyte growth factor, which have all been shown to promote corneal epithelial wound healing (Mead et al., 2020). It can either be performed in a single or multilayer (for deep ulcers with stromal tissue loss), with either in-lay or over-lay techniques used. In the in-lay technique, the AM is applied as a permanent basement membrane substitute and is sutured with the epithelial side facing outwards, allowing recipient epithelial cells to migrate onto the AM. An AM in-lay will be permanently incorporated and remodeled into the host cornea (Seitz et al., 2006). In the over-lay technique, the AM acts as a ‘patch’ or biological ‘bandage’ and is sutured with the epithelial side facing inwards, and will detach from the corneal surface after 1-2 weeks. (Meller et al., 2011). The sandwich technique is a combination of both the in-lay and over-lay techniques.

Corneal neurotization (CN) has is a potentially curative surgical procedure for NK (Terzis et al., 2009). Two main surgical approaches have been described. Direct CN (DCN) involves the transposition of the contralateral or ipsilateral supraorbital/supratrochlear nerves to the anesthetic cornea, whilst indirect CN (ICN) involves interposition of a nerve graft (typically the sural nerve) between the supraorbital and/or supratrochlear nerves and the affected cornea (Fogagnolo et al., 2020). A prospective comparative series comparing DCN and ICN showed that there was no difference between the groups: NK was healed in all patients after a mean period of 3.9 months, mean corneal sensitivity improved from 3.07 to 22.11 mm (*p* < 0.001), and a corneal sub-basal nerve plexus was detectable in all eyes (Fogagnolo et al., 2020). Catapano et al. (Catapano et al., 2019) reported on 19 eyes which had ICN, with a significant improvement in mean central corneal sensitivity from 0.8 ± 2.5 mm to 49.7 ± 15.5 mm at final follow-up (*p* < 0.001). The number of episodes of corneal epithelial defects after MICN was significantly reduced compared with the year leading up to the procedure (21 vs. 89%; *p* < 0.0001).

## Conclusion

NK is a challenging disease to treat, and patients often lose vision. The aim of therapy is to treat any underlying causative factors and to promote epithelial healing, whilst preventing progression of disease. The emergence of rhNGF and NGF mimetics hold exciting and promising possibilities, and may be useful in cases of NK that are refractory to conventional non-specific treatment.
